# Worms

**Published:** 1891-09

**Authors:** 


### WORMS.

One of the most valuable works of Mr. Darwin was on Worms. Probably he was among the first that drew the world's attention to their immense service. In a certatn district in West Africa earth worms are astonishingly abundant, and the richness of the soil is largely attributed to their work. It has been estimated that within the radius of a single square mile not less than 62,223 tons of subsoil are brought to the surface by them annually, and that within a period of twenty-seven years they will bring to the surface all the soil for a depth of two feet. This is one of the progresses of nature's God for the recovery of the strength of the soil. His servants are found in all forms of His creation, and He adapts all the forms of life to labor, and assigns them to their work.

